# Functional Groups of Summer Copepod Assemblage in the Central Barents Sea

**DOI:** 10.3390/ani16213405

**Published:** 2026-10-28

**Authors:** Vladimir G. Dvoretsky, Alexander G. Dvoretsky

**Affiliations:** Murmansk Marine Biological Institute of the Russian Academy of Sciences (MMBI RAS), Murmansk 183038, Russia

**Keywords:** plankton, copepods, Arctic shelf, spatial variability, functional traits

## Abstract

The functional trait structure of copepod assemblages inhabiting Arctic seas remains poorly investigated. In the present study, we aimed to reveal the spatial pattern of copepod functional groups in the central Barents Sea. One type of water mass (the Barents Sea Water) was identified based on an analysis of water properties. Environmental gradients in oceanographic factors, nutrients, and food conditions were relatively stable. Total abundance ranged from 434 to 1168 ind. m^−3^, with *Oithona similis* dominated numerically. Total biomass ranged from 7.1 to 85.9 mg dry mass m^−3^, with *Calanus* copepods having the highest contribution. Four copepod groups were delineated based on seven key functional traits (size, trophic group, feeding mode, reproductive strategy, life cycle duration, preferred habitat, and geographic distribution). The most abundant group included *Calanus finmarchicus*, *Pseudocalanus* spp., *Microcalanus* spp., *Centropages hamatus*, and Copepoda nauplii, which attained high abundances in the northern and south-eastern sub-regions. RLQ, fourth-corner, and redundancy analyses indicated sampling depth as the most important variable explaining spatial variation in diversity, functional diversity, and copepod traits. Water temperature, chlorophyll *a* concentration, and nutrient levels had a significant impact on particular functional traits, including omnivore–herbivores, omnivores, cruise-feeding, mixed-feeding, long-lived, intermediate-depth dwellers, and Arctic/cosmopolitan taxa. This research provides baseline information on the functional trait structure of copepod populations in the highly productive Arctic region and can be applied to the future monitoring of pelagic assemblages.

## Simple Summary

Copepods are the dominant components of zooplankton communities in Arctic seas. The Barents Sea is the largest Arctic shelf region, and its summer productivity depends heavily on plankton abundance. This study examined the functional structure of copepod communities within the Barents Sea Water mass. The composition, structure, and distribution of copepods showed high stability in quantitative characteristics. Functional traits were found to be linked to bathymetric conditions. Several environmental factors also played important roles in shaping the trophic and size structure of copepod populations. This work enhances our understanding of pelagic community organization in Arctic seas and provides a foundation for future research on plankton responses to climate variability.

## 1. Introduction

Arctic marginal seas are dynamic ecosystems supporting diverse and abundant pelagic organisms that play a significant role in the functioning of carbon cycling [1]. The Barents Sea is considered to be one of the most productive shelf regions, hosting numerous and rich marine biotic communities and extensive fisheries that are crucial for the economies of Russia and Norway [2]. The region is a transitional zone between the North Atlantic and the Arctic Oceans [3]. Environmental conditions vary strongly, with the coldest areas in the north where ice cover persists from autumn to late spring [4]. The southern areas are affected by warm currents from the Norwegian Sea and may be ice-free throughout the year. The central part has a clear boundary between the Atlantic Water and the Arctic Water, and, here, the Polar Front separates cold and warm regions [5]. The Barents Sea Water is formed as a result of the cooling of Atlantic Water and occupies a significant part of the central and eastern regions during summer periods. The Coastal Water is a diverse water mass that exhibits some modifications in relation to geographic position—Murmansk Coastal Water, Novaya Zemlya Coastal Water, Pechora Sea Coastal Water, and Svalbard Coastal Water [5]. Climatic conditions in the Barents Sea show marked seasonal and interannual variability, reflecting changes in global climatic and atmospheric processes [6].

In the Arctic, a warming trend has been documented since the end of the 20th century, which has led to a drastic decline in sea ice cover, a prolonged ice-free period, rising air and water temperatures, and changes in the frequency of storms and extreme meteorological phenomena [7,8]. In addition, strong intrusions of Atlantic Water have caused a northward shift in the Polar Front [9]. The past decade has been identified as the warmest period in the Barents Sea, and this has led to shifts in the biotic structure of pelagic and benthic communities [10,11]. Primary production has increased due to an earlier spring bloom and a prolonged period of phytoplankton activity [12,13].

Zooplankton represents a key link connecting primary producers to higher trophic levels [14,15]. Arctic planktonic animals demonstrate clear seasonality in relation to varying environmental conditions and reach their peak in the spring and summer periods, during or after the main maximum of phytoplankton abundance [16,17,18,19,20,21,22]. Copepods are considered the most important mesozooplankton group, accounting for a major part of the total zooplankton abundance and biomass in the Arctic Ocean and adjacent seas [23,24,25,26,27,28]. The production of copepods is utilized by macrozooplankton, micronekton, and pelagic fish, thus determining the biomass and production of higher trophic levels, including seabirds and marine mammals [14]. Moreover, copepod biomass and stocks may be used as good predictors for commercial stocks of important fish, including both pelagic (capelin and herring) and demersal (cod and haddock) species [29,30]. Research concerning copepod ecology in the Barents Sea is extensive, but most studies have focused on spatial patterns of abundance, biomass, and diversity, as well as on the biology of common taxa (*Calanus*, *Oithona*, *Pseudocalanus*, etc.) [19,20,21,31,32,33,34,35,36]. However, little is known about the functional traits of zooplankton in the Barents Sea and other Arctic regions [37,38].

A functional approach is considered one of the most effective ways to characterize planktonic fauna in aquatic ecosystems [39]. This method allows copepods to be classified according to their functional traits [40,41,42,43]. Functional traits can include a range of characteristics, among which ecological, behavioral, morphological, physiological, and biogeographic traits are the most important [44]. All these features define the ecological position and role of any species in a given community and reflect the interactions of a taxon with other organisms and with the environment [45]. The relative proportions of functional groupings may underlie the structure and functioning of aquatic ecosystems, thereby determining ecological balance and carbon cycling in a particular region. A functional group encompasses a community of taxa with similar traits that exert similar impacts on ecosystem processes. Therefore, it is expected that they will demonstrate similar responses to a suite of ecological factors [46]. Thus, a functional trait-based approach is assumed to be a better method for describing ecological complexity than a purely taxonomic approach, because it provides a detailed ecological description of ecosystem functioning without the need to study each species separately. This approach has been applied more widely only in recent years, as species-specific trait information has become available. This is especially true for copepods, owing to the systematization of information on copepod traits in special databases [47]. There are several good examples demonstrating the advantages of applying a functional trait-based approach to marine copepod and zooplankton investigations [40,46,48,49,50]. While a recent study by Durazzano et al. [38] investigated a similar topic in the northwestern Barents Sea, our research extends this scope to a previously unexamined region of the sea. Consequently, our work makes two key contributions: it is the first to investigate the functional traits of copepods in this specific, highly productive area of the Arctic, and it provides the first comprehensive assessment of the functional diversity of planktonic organisms across the central Barents Sea.

The present study focuses on a region located in the central Barents Sea. The area is occupied by Barents Sea Water, which is the main type of water mass in this region. Spatial variability in mean temperature and salinity is relatively low, although vertical gradients may be present. The goals of the research were to: (1) describe the spatial pattern of copepod abundance, biomass, and diversity; (2) identify functional groups of copepods; (3) estimate functional diversity indices of the copepod assemblage; and (4) assess the relationships between functional groups and environmental factors. We tested the hypotheses that (1) the copepod community would be relatively similar within the study area owing to weak horizontal gradients in oceanographic properties, and (2) the diversity and functional diversity of the copepod assemblage would be affected by environmental conditions in the region.

## 2. Material and Methods

### 2.1. Sampling

**Figure 1:**
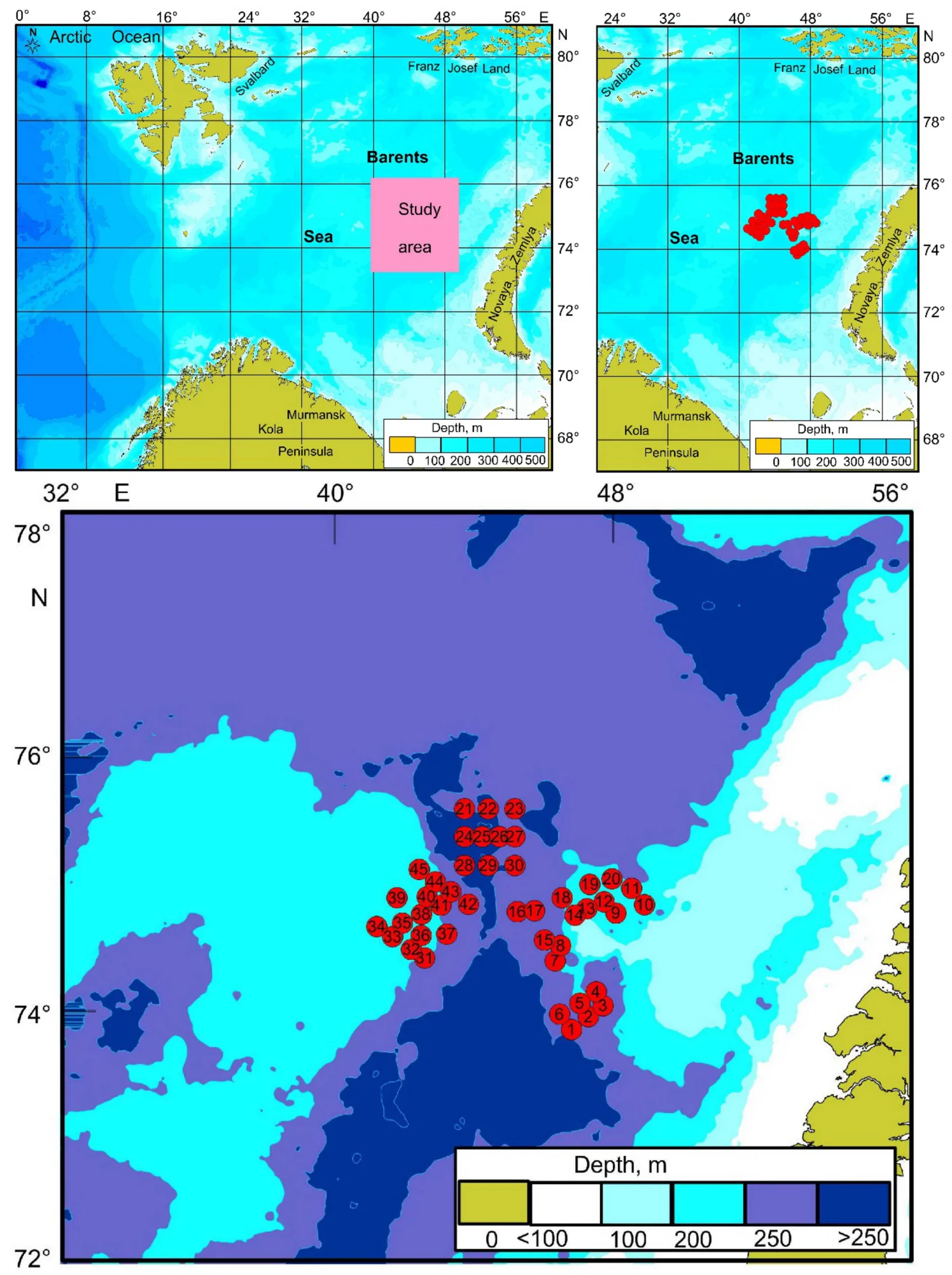
Map of copepod sampling stations in the central Barents Sea, summer 2015, with detailed bathymetry (**lower**).

Zooplankton samples were collected in the central Barents Sea within the framework of multidisciplinary investigations of Murmansk Marine Biological Institute (MMBI) during a cruise of RV *Dalnie Zelentsy* in July 2015 (Figure 1, Table S1). A Juday net (0.11 m^2^ mouth opening, mesh size 168 μm) equipped with a flowmeter was hauled vertically from near the bottom (5–7 m) to the surface twice at 45 stations. The net was hauled at a speed of 1 m s^−1^ to ensure adequate water filtration [51]. The sampling sites were located in the Barents Sea Water, the main water mass that is formed as a result of cooling of the Atlantic Water. Water temperature and salinity were measured before the zooplankton net sampling using an oceanographic conductivity–temperature–depth profiler (Sea-Bird Scientific, Bellevue, Washington, DC, USA). Samples to determine chlorophyll a (Chl-a) concentrations and for hydrochemical analyses were collected from the surface, pycnocline, and bottom layers using a rosette sampler equipped with 10 L Niskin bottles (Hydro-Bios, Altenholz, Germany).

### 2.2. Processing

Chl-a concentration was determined using a standard protocol: 2 L of seawater were filtered onto Vladiopore filters (0.6 μm) (Science-Technical Center Vladiopor, Vladimir, Russia), and the phytopigments were then extracted in 5 mL of 96% ethanol. The absorbance was measured using a Nicolett Evolution 500 spectrophotometer (Spectronic Unicam, Cambridge, UK) following the standard procedure [52]. Dissolved oxygen concentrations were determined using the Winkler titration method [53]. Analyses of dissolved inorganic nutrients (nitrate, nitrite, phosphate, and silicate) were carried out at the MMBI laboratory according to standard methods, using a PE-5300-VI spectrophotometer (Ecos-Analitika LLC, St. Petersburg, Russia) [54]. Prior to analysis, the spectrophotometers were calibrated against standards provided by the manufacturers.

In the laboratory, zooplankton samples were split into subsamples comprising no less than 1/10 of the total sample and encompassing 300–1000 individuals. All copepods were sorted, identified to the lowest possible taxonomic level, and enumerated using an MBS-10 stereomicroscope at 4–56× magnification. Copepod abundance was calculated taking into account the total volume of water filtered during sampling and was converted into individuals per cubic meter (ind. m^−3^). Developmental stages of *Calanus* spp. and adults and copepodite stage V of *Pseudocalanus* spp. were separated by prosome length and morphological criteria [55,56]. Individual biomass was estimated using length–mass equations and published data on mean individual wet, dry, or carbon mass [57,58,59,60]. All values were computed as mg dry mass (DM) per cubic meter (mg DM m^−3^). To convert wet and carbon mass to dry mass, two relationships were used: 1 mg wet mass = 0.2 mg DM and 1 mg DM = 0.4 mg C [51]. Total copepod biomass was calculated as the sum of the biomasses of particular taxa. To create contour maps of abundance and of functional groups, we used data integrated for the whole water column (10^3^ ind. m^−2^).

Functional traits for copepods were obtained from published sources [40,41,48,61,62,63]. Seven crucial functional traits were used in this study (Table 1 and Table S2): size, trophic mode, feeding mode, reproductive strategy, life span, preferred habitat, and geographic distribution. The functional traits applied were selected to indicate important ecological and biological characteristics of the copepod taxa, including their mass, reproductive potential, grazing impact on phytoplankton, predator–prey interactions within the zooplankton assemblage, position in food webs, ecological niche, and associations with warm and cold habitats [41,44]. To ensure transparency, we treated mixed and developmental categories as follows. Copepoda nauplii were assigned traits based on the dominant calanoid nauplii in the study area, primarily *Calanus* spp. and *Pseudocalanus* spp., because these taxa dominate naupliar assemblages in the central Barents Sea. *Pseudocalanus* spp. I–IV were assigned the traits of the genus *Pseudocalanus*, following published trait matrices for marine copepods [40,48,62]. *Microcalanus* spp. and other genus-level categories were similarly assigned using congeneric trait information. When a category contained multiple possible trait modalities, the modality most consistently reported for the dominant constituent taxa was used. These categories were retained in the main analysis because they represent a substantial part of copepod abundance in the region.

### 2.3. Statistics

Environmental conditions were assessed using principal component analysis (PCA) based on the correlation matrix. PCA was applied to identify spatial separation among the sampling stations. One-way PERMANOVA (based on Euclidean distance) was used to detect differences between groups of stations delineated with PCA. PERMANOVA involved 999 permutations to calculate *p*-values.

Copepod community structure was investigated using univariate and multivariate analyses [64]. The number of taxa, species dominance (the inverse Simpson index), Shannon diversity, and Pielou evenness were calculated to quantify copepod diversity [65]. The functional indices estimated were: FDis—functional dispersion, FMPD—functional mean pairwise distance, FNND—functional mean nearest neighbor distance, FEve—functional evenness, FRic—functional richness, FDiv—functional divergence, FOri—functional originality, and FSpe—functional specialization [66,67,68,69]. FRic, FEve, and FDiv are considered analogs of taxonomic diversity indices.

Prior to the multivariate analyses, copepod abundance data were log-transformed, and a matrix based on the Bray–Curtis similarity index was constructed. A hierarchical clustering method was applied to visualize the data. One-way PERMANOVA was performed to assess spatial differences in copepod abundance between sampling locations [64]. Copepod functional groups were delineated based on the seven functional traits. The trait classification for each taxon was presented as a binary matrix coded 0 or 1. Taxa possessing a given trait modality were assigned a value of 1, whereas taxa lacking the modality were assigned a value of 0. Hierarchical cluster analysis was applied to create a dendrogram showing the separation of functional groups (Gower distance, Ward’s method), using the factoextra package (version: 2.2.0) in R (version 4.0.0) [70]. Gower’s distance was selected as the dissimilarity measure due to the mixed nature of the dataset, which included categorical predictors. This metric computes the partial dissimilarity for each variable independently, applying appropriate normalization to each feature type, and subsequently aggregates them into a single coefficient by averaging. Furthermore, the Gower metric inherently accommodates missing values, which were present in our dataset, without requiring prior imputation. Ward’s method was employed for hierarchical agglomerative clustering. This algorithm seeks to minimize the increase in the within-cluster sum of squares (inertia) at each fusion step. This analytical framework is essential for investigating the functional structure of biological communities. The objective is to ensure that taxa (species) with similar combinations of functional traits are grouped within the same cluster, thereby producing well-defined, discrete clusters with clear trait-based differentiation between them. The optimal number of clusters was determined using the Ptbiserial index [71].

To explore covariation between copepod functional traits and environmental variables, we performed an RLQ analysis [72]. The RLQ method allows investigation of the joint structure among three tables: the R-table (environmental variables), the Q-table (species traits), and the L-table (species abundances) [72]. Table L measures the intensity of the associations between the R-table and Q-table. The best joint combination of the ordination of sites by their environmental characteristics, the biplot ordination of copepod taxa by their traits, and the simultaneous ordination of taxa and sites was calculated. Thus, the RLQ analysis provides three separate plots showing covariance between traits and the environmental dataset. We then performed a fourth-corner analysis [73], which tests for individual correlations between copepod traits and environmental variables. Variance inflation factors (VIF) were used to detect multicollinearity among environmental variables, and predictors with VIF > 3 were excluded from the analyses [64]. Final input variables were non-transformed sampling depth, surface temperature, surface salinity, bottom salinity, mean Chl-a, surface phosphate, surface nitrite, surface nitrate, mean nitrate, and surface silicate. The fourth-corner analysis was used to detect the significant trait–environment associations. To evaluate the robustness of the identified trait–environment relationships, we conducted a sensitivity analysis by excluding Copepoda nauplii and *Pseudocalanus* spp. I–IV. We quantified the structural similarity between the matrices of fourth-corner statistics from the full and reduced datasets using the RV coefficient. The RV coefficient serves as a multivariate generalization of the squared Pearson correlation coefficient (R^2^), providing a measure of the global concordance between two matrices. The statistical significance of the matrix similarity was assessed using a Monte-Carlo permutation test (999 permutations) implemented in the *ade4* package (version: 1.7-24).

Indicator species of copepods in different functional groups were detected using the indicator value (IndVal) analysis [74]. High IndVal values indicate an association of particular taxa with specific functional groups, whereas low IndVal values suggest a more uniform distribution of taxa across all functional groups.

Redundancy analysis (RDA) was used to explore environmental drivers of diversity and functional diversity metrics. Separate RDAs were performed independently on the diversity dataset (species richness, the inverse Simpson index, Shannon index, and Pielou evenness) and the functional diversity dataset (FDis, FMPD, FNND, FEve, FRic, FDiv, FOri, and FSpe). Input explanatory variables were the non-collinear environmental variables retained in the RLQ analysis. Forward selection of environmental variables for the RDA models was performed using a Monte Carlo permutation test (*n* = 9999) [75] and a stopping criterion of *p* < 0.05. At each step, the variable explaining the largest amount of additional variance was added only if its conditional (marginal) effect remained significant after permutation testing. To reduce the risk of inflated significance from testing multiple explanatory variables, we applied a Bonferroni-type correction to the selection threshold. Variables with variance inflation factors (VIF) > 3 were excluded before selection to avoid multicollinearity. Forward selection can overestimate the importance of individual predictors when many correlated variables are tested, and we therefore interpret the selected variables as correlates rather than confirmed causal drivers. Diversity metrics, cluster analysis, and PCA were performed using PAST 4.14. Descriptive statistics, and significance tests were done with STATISTICA 10.0. Functional diversity indices and RLQ were computed in the open-source software R 4.3.1 [76] using the *ade4* (version: 1.7-24) and *mFD* packages (version: 1.0.7). RDA models were performed using Canoco 4.5. Mapping of environmental and biotic variables was performed in MapViewer 8.1 (Golden, CO, USA). Contour maps were produced using kriging as the interpolation procedure (automatic function in MapViewer 8.1).

## 3. Results

### 3.1. Environmental Background

**Figure 2:**
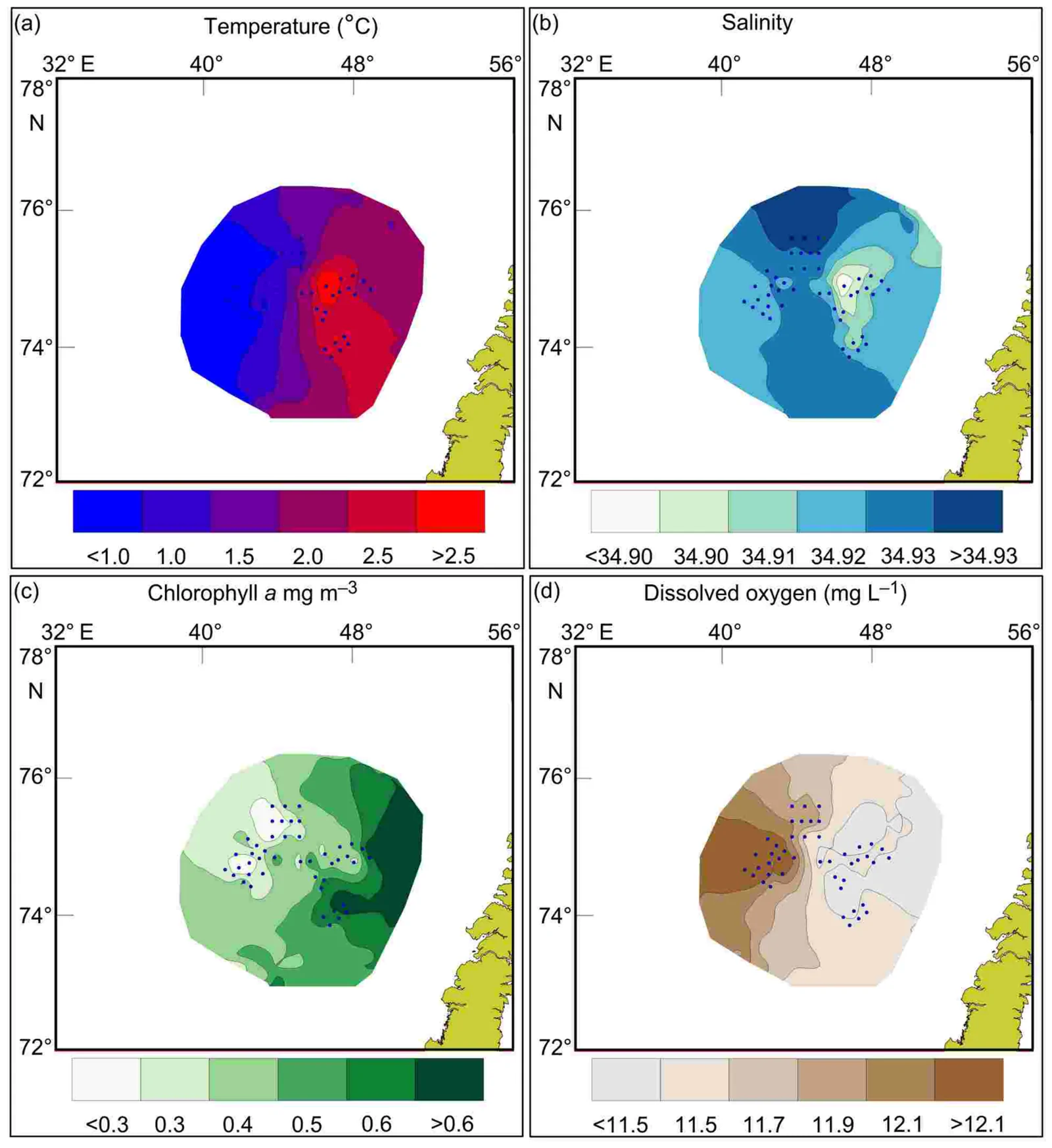
Spatial distribution maps of mean surface water properties in the central Barents Sea, summer 2015: (**a**) temperature (°C), (**b**) salinity, (**c**) chlorophyll *a* concentration (mg m^−3^), and (**d**) dissolved oxygen concentration (mg L^−1^). Values represent depth-averaged concentrations within the sampling layer.

**Figure 3:**
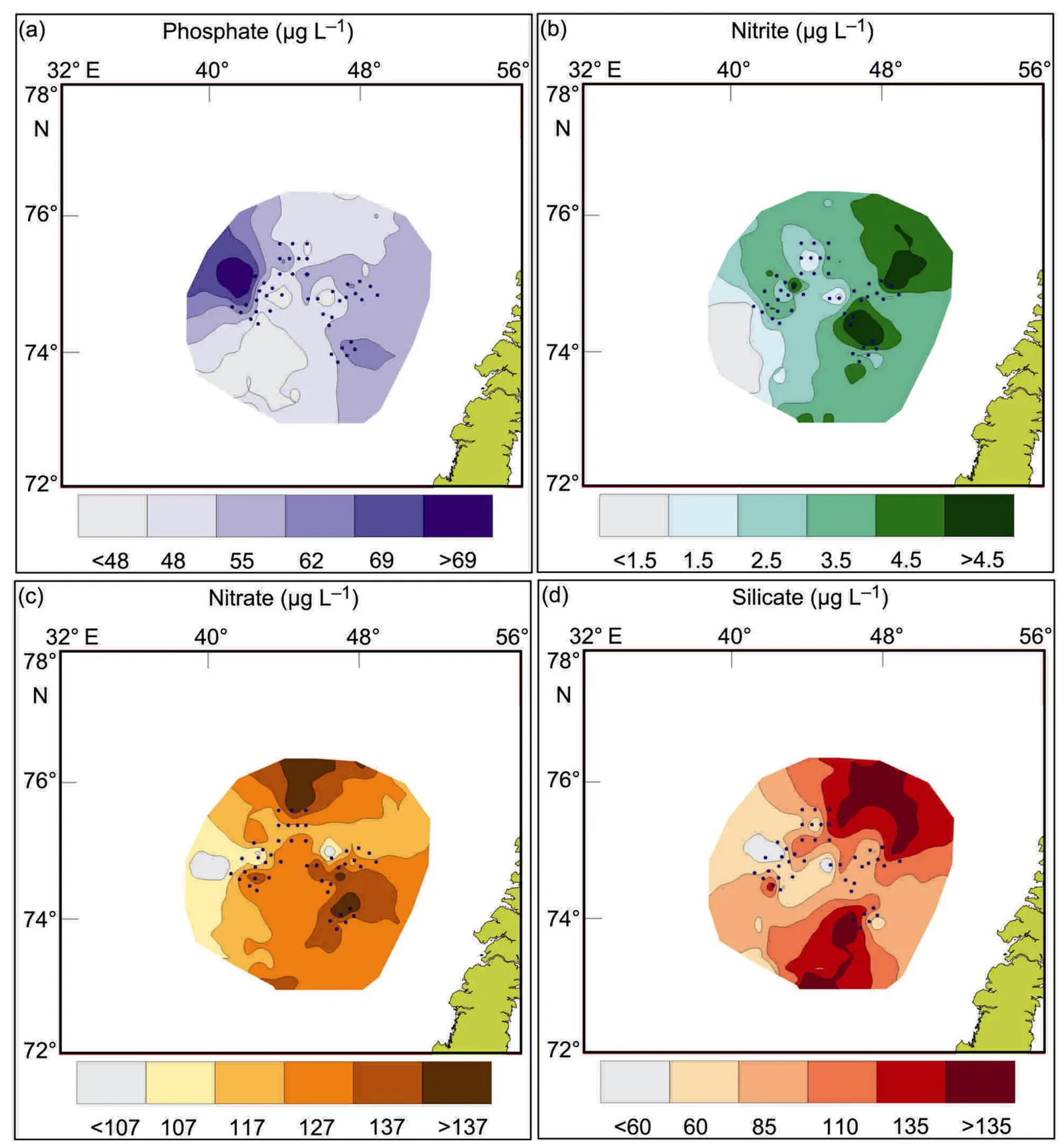
Spatial distribution maps of mean nutrient concentrations in the central Barents Sea, summer 2015: (**a**) phosphate (µg L^−1^), (**b**) nitrite (µg L^−1^), (**c**) nitrate (µg L^−1^), and (**d**) silicate (µg L^−1^). Values represent depth-averaged concentrations within the sampling layer.

Environmental factors in the water column varied among the sampled stations (Figure 2). Principal component analysis revealed two regions. The first region included stations 1–20, 23, and 29–30, and the second encompassed stations 21–22, 24–28, and 31–45. Cluster analysis also revealed two groups of stations, which showed a high degree of similarity (75%). PERMANOVA detected no significant differences between the regions identified by PCA (F = 1.200, *p* = 0.288). Therefore, we analyzed environmental and biotic datasets as combined variables for all stations. Mean water temperature tended to increase from west to east (Figure 2a). Mean salinity was relatively stable in the study area (Figure 2b). Mean Chl-a concentrations were highest in the eastern part (Figure 2c), whereas the dissolved oxygen maximum was recorded in the west (Figure 2d). The same pattern as dissolved oxygen was detected for phosphate (Figure 3a). Nitrite and silicate concentrations reached their maxima in the north-eastern part (Figure 3b,d). Nitrate concentrations were high in the north (Figure 3c). Table 2 summarizes environmental conditions in the study area.

### 3.2. Copepod Abundance and Biomass

Total copepod abundance varied widely from 434 to 1168 ind. m^−3^, averaging 734 ± 190 ind. m^−3^. The small copepod *Oithona similis* dominated numerically and accounted for 30% of the entire assemblage. The oceanic species *Calanus finmarchicus* contributed 17% of the abundance in the region. *Pseudocalanus* spp., *Metridia longa*, and Copepoda nauplii made up 15%, 14%, and 13% of the copepod assemblage by numbers, respectively (Table 3). Total copepod biomass ranged from 7.1 to 85.9 mg DM m^−3^, with a mean of 38.5 ± 18.9 mg DM m^−3^. Large and medium-sized copepods of the genus *Calanus* constituted the main part of the copepod biomass (80%), with *C. glacialis* having the highest contribution (39%). The mean share of *M. longa* was also relatively high (17%) (Table 3). Copepod abundance and biomass demonstrated opposite spatial patterns: total abundance reached its maximum in the southern part of the study area, whereas total copepod biomass was highest in the north.

### 3.3. Functional Groups of Copepods

**Figure 4:**
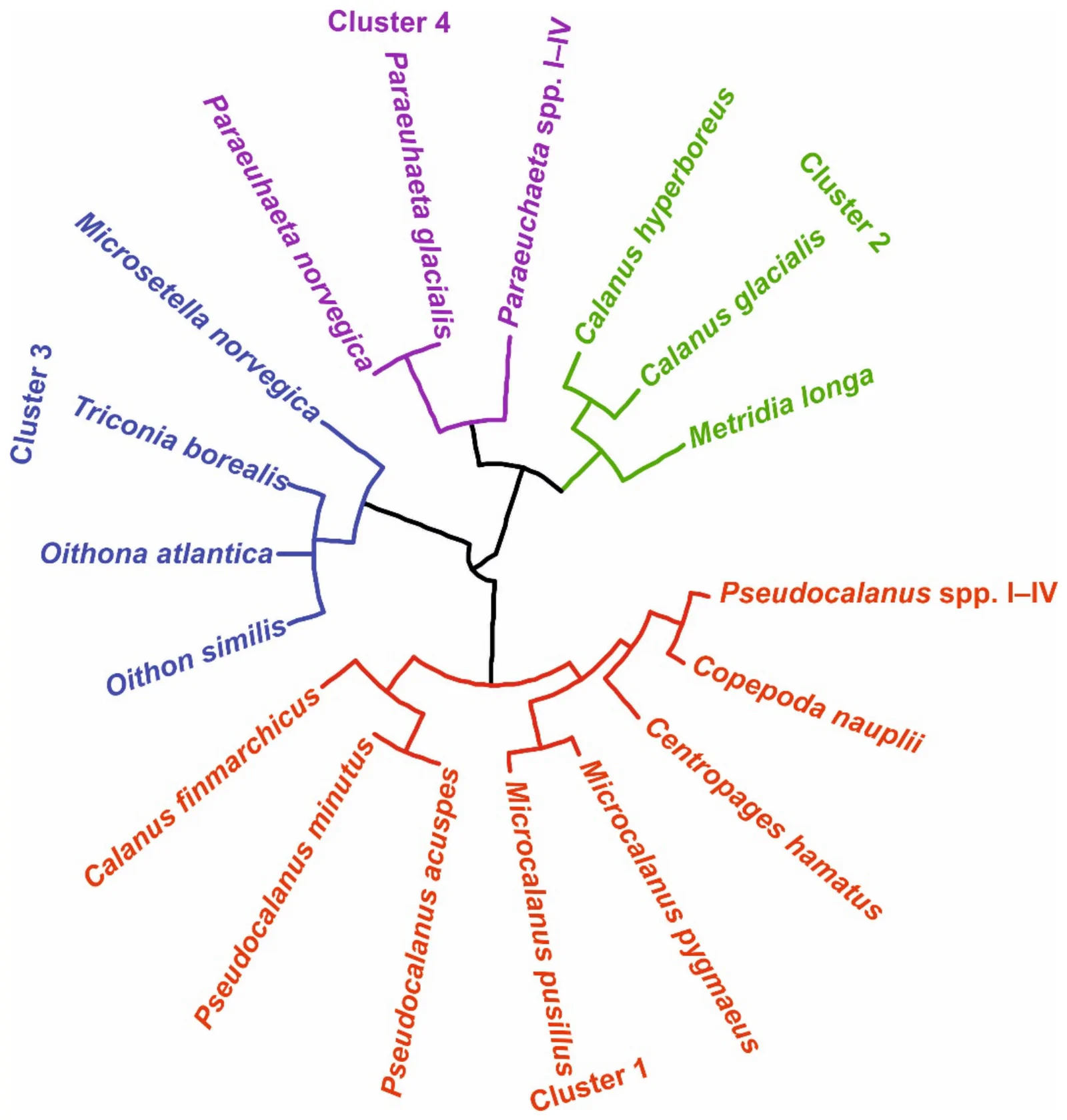
Results of cluster analysis showing separation of copepods based on their functional traits in the central Barents Sea, summer 2015.

**Figure 5:**
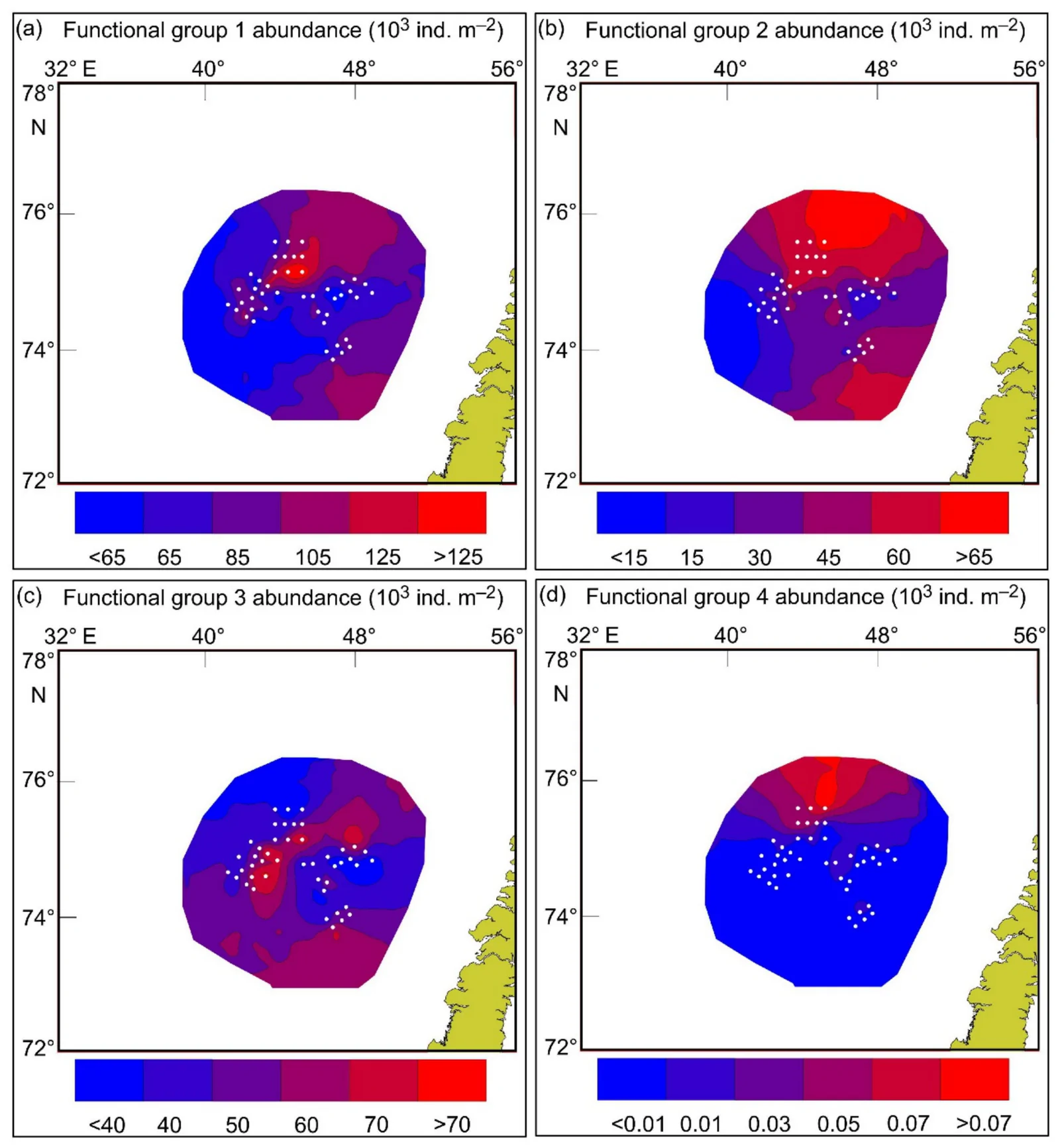
Spatial distribution of total integrated abundance of copepod functional groups in the central Barents Sea, summer 2015.

Cluster analysis and IndVal analysis revealed four functional groups of copepods based on their functional traits (Figure 4). The first group included boreal, boreal–Arctic, and cosmopolitan species *Calanus finmarchicus*, *Pseudocalanus* spp., *Microcalanus* spp., *Centropages hamatus*, and Copepoda nauplii. These taxa were characterized by a prevailing omnivore–herbivore trophic strategy, a feeding-current mode, relatively short life cycles, and occurrence mainly in the upper water layer. The total integrated abundance of group 1 attained high values in the northern and south-eastern sub-regions (Figure 5a). The second group comprised *Calanus glacialis*, *Calanus hyperboreus*, and *Metridia longa*, which typically dominate in Arctic Water. These large, broadcast-spawning species have longer life cycles (>1 year). The total integrated abundance of group 2 demonstrated the same spatial pattern as group 1 although it was numerically less abundant than the first group (Figure 5b). The small species *Microsetella norvegica*, *Oithona atlantica*, *Oithona similis*, and *Triconia borealis* were combined into group 3. These taxa are cosmopolitan, sac-spawning, active ambush-feeding species that have short life cycles and form more than one generation per year. Small taxa dominated mainly in the southern and central parts of the study area (Figure 5c). The fourth group encompassed large, long-lived *Paraeuchaeta* species, which are classified as sac-spawning species with a carnivorous trophic mode. These taxa inhabit intermediate/deep, cold waters in the north (Figure 5d). The total integrated abundance of group 4 was the lowest among all copepod functional groups (Figure 5d).

### 3.4. Taxonomic Diversity and Functional Diversity

**Figure 6:**
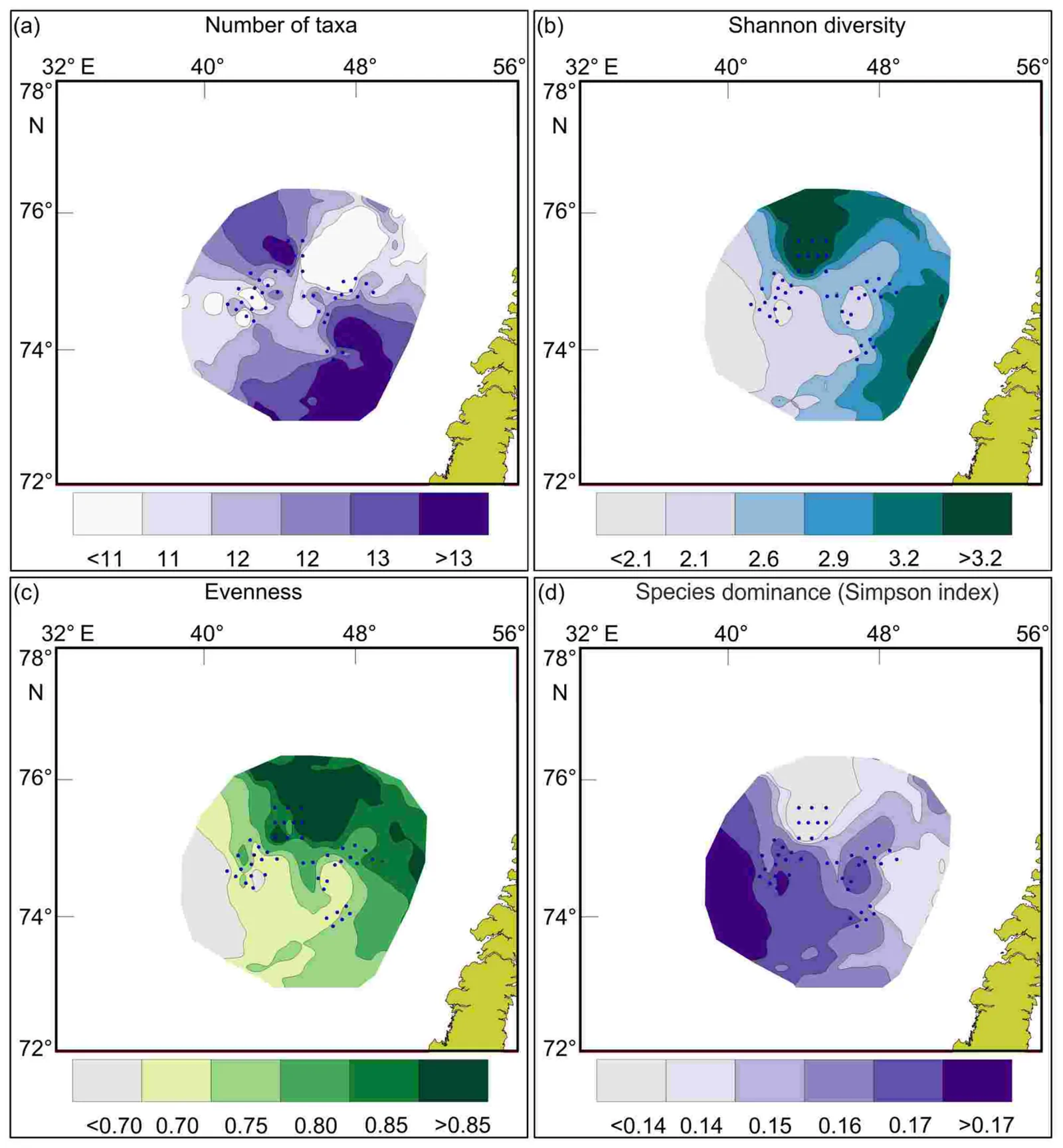
Spatial patterns of taxonomic diversity indices for the copepod assemblage in the central Barents Sea, summer 2015: (**a**) taxa richness, (**b**) Shannon diversity (H’), (**c**) Pielou’s evenness, and (**d**) the inverse Simpson index.

The number of taxa varied from 12 to 16 and tended to be highest in the south-eastern part of the research area (Figure 6a). The Shannon–Wiener index and Pielou evenness ranged from 2.09 to 3.19 and from 0.56 to 0.87, respectively, and reached their maximal values in the northern sub-region (Figure 6b,c). Species dominance, estimated using the inverse Simpson index, was 1.56–2.30 and reached its maximum in the south-western part of the study area (Figure 6d).

**Figure 7:**
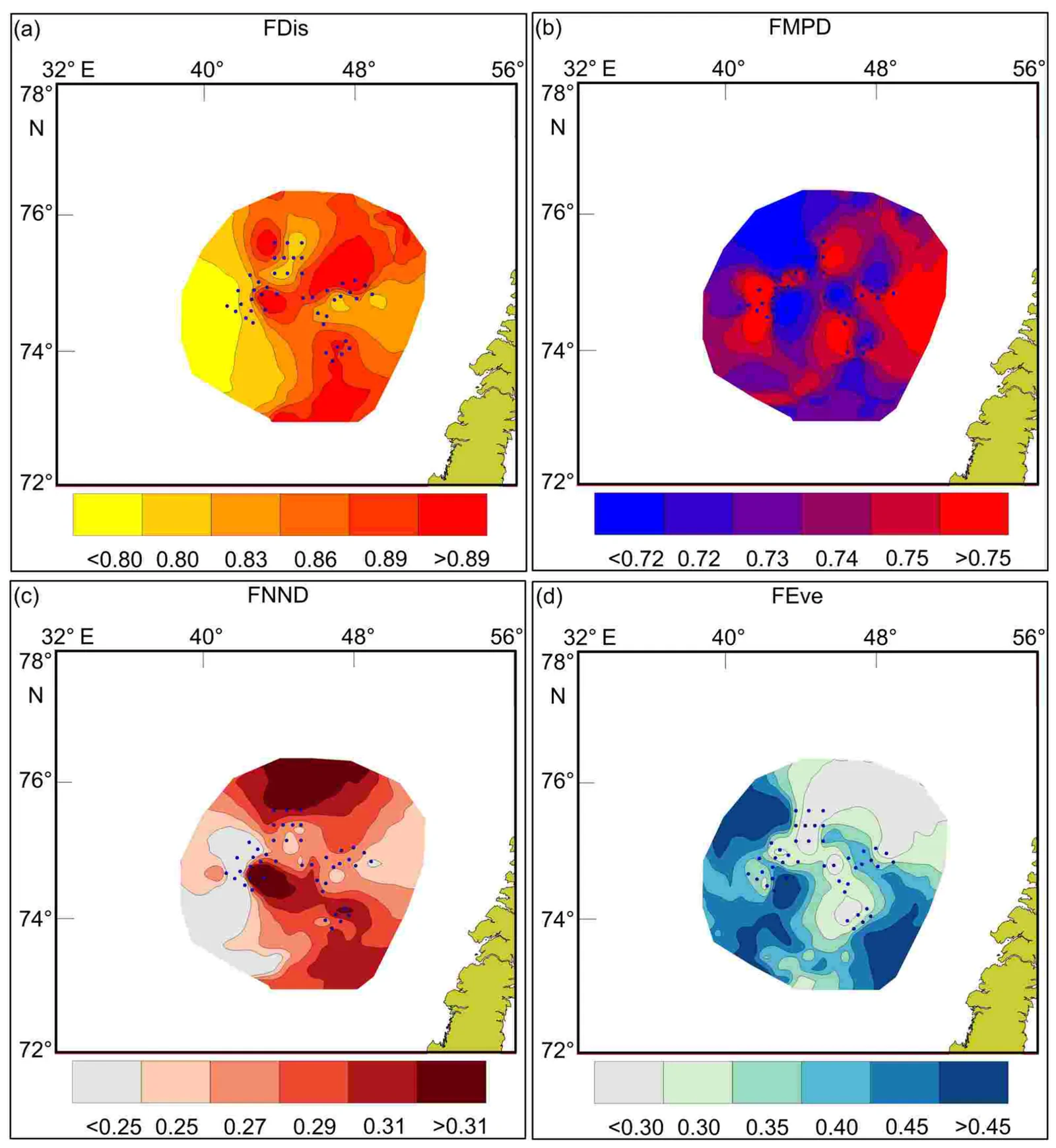
Functional diversity indices of the copepod assemblage in the central Barents Sea, summer 2015. FDis—functional dispersion, FMPD—functional mean pairwise, FNND—functional mean nearest neighbor distance, FEve—functional evenness.

**Figure 8:**
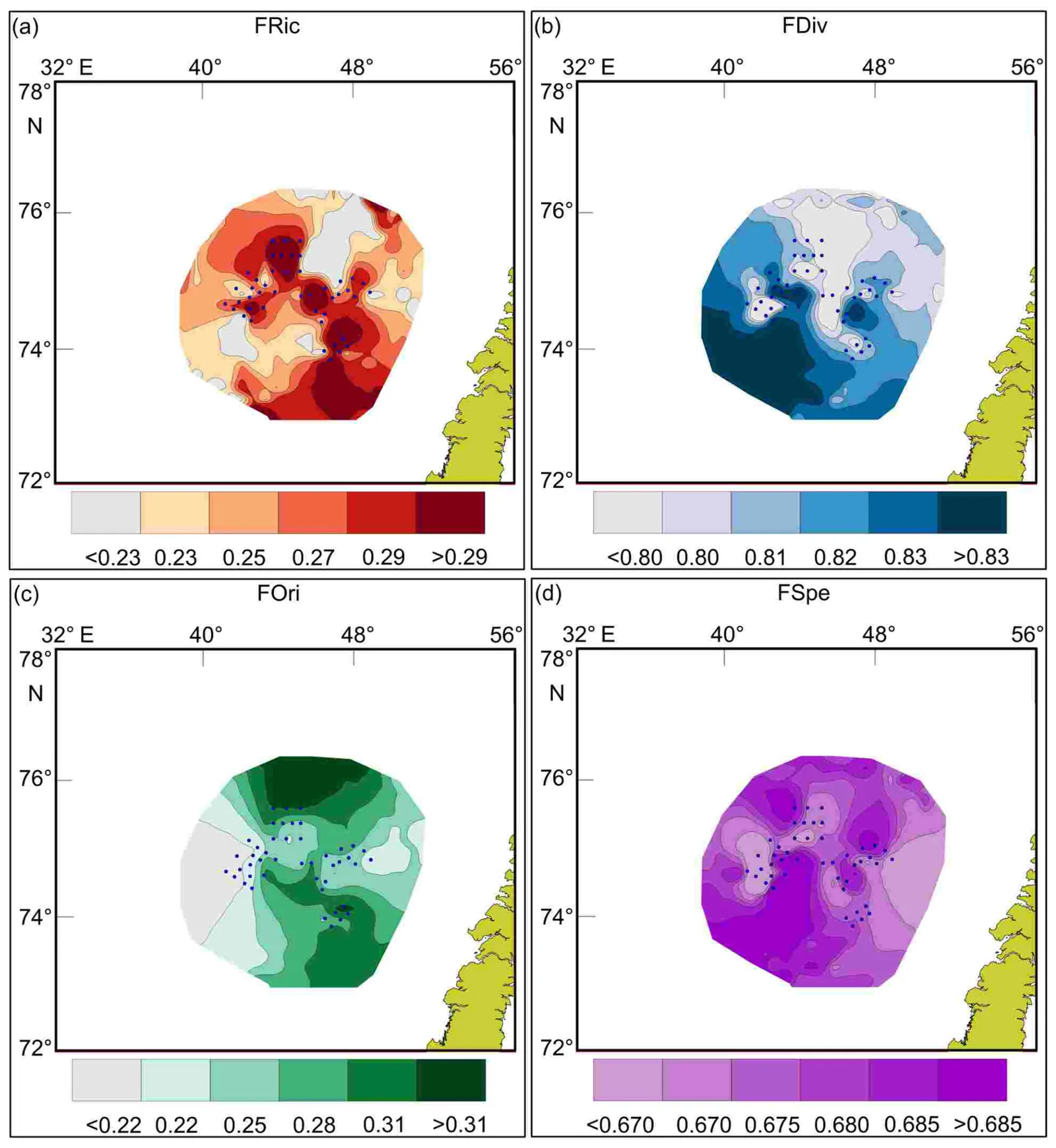
Functional diversity indices of the copepod assemblage in the central Barents Sea, summer 2015. FRic—functional richness, FDiv—functional divergence, FOri—functional originality, FSpe—functional specialization.

Functional dispersion of the copepod assemblage varied between 0.76 and 0.87, with maximal values in the central and southern parts (Figure 7a). Functional mean pairwise distance attained high values in the east and ranged from 0.71 to 0.79 (Figure 7b). Functional mean nearest neighbor distance exhibited two hotspots in the northern and central parts of the study area, and this metric varied from 0.19 to 0.42 (Figure 7c). Functional evenness ranged from 0.01 to 0.65, with two maxima in the west and south-east (Figure 7d). Functional richness showed high variability (0.13–0.98), with the highest values in the central and south-eastern sub-regions (Figure 8a). Functional divergence tended to increase from the north-east to the south-west, from 0.78 to 0.89 (Figure 8b). Functional originality attained its maximal values in the north (0.34), whereas the lowest values were recorded in the east and west (Figure 8c). Functional specialization varied only slightly across the study area (0.64–0.70), with relatively high values in the central and south-western sub-regions (Figure 8d).

### 3.5. Associations of Copepod Traits, Diversity, and Functional Diversity with Environmental Variables

The first two RLQ axes explained 94.4% of the total variation (Axis 1: 75.5%, Axis 2: 18.9%). The randtest indicated that the RLQ analysis was significant at *p* < 0.05. The first RLQ axis was significantly correlated with sampling depth, surface water temperature, and surface phosphate concentration. The second axis showed strong correlations with mean Chl-a, surface salinity, temperature, bottom salinity, and surface phosphate concentration (Table 4).

**Figure 9:**
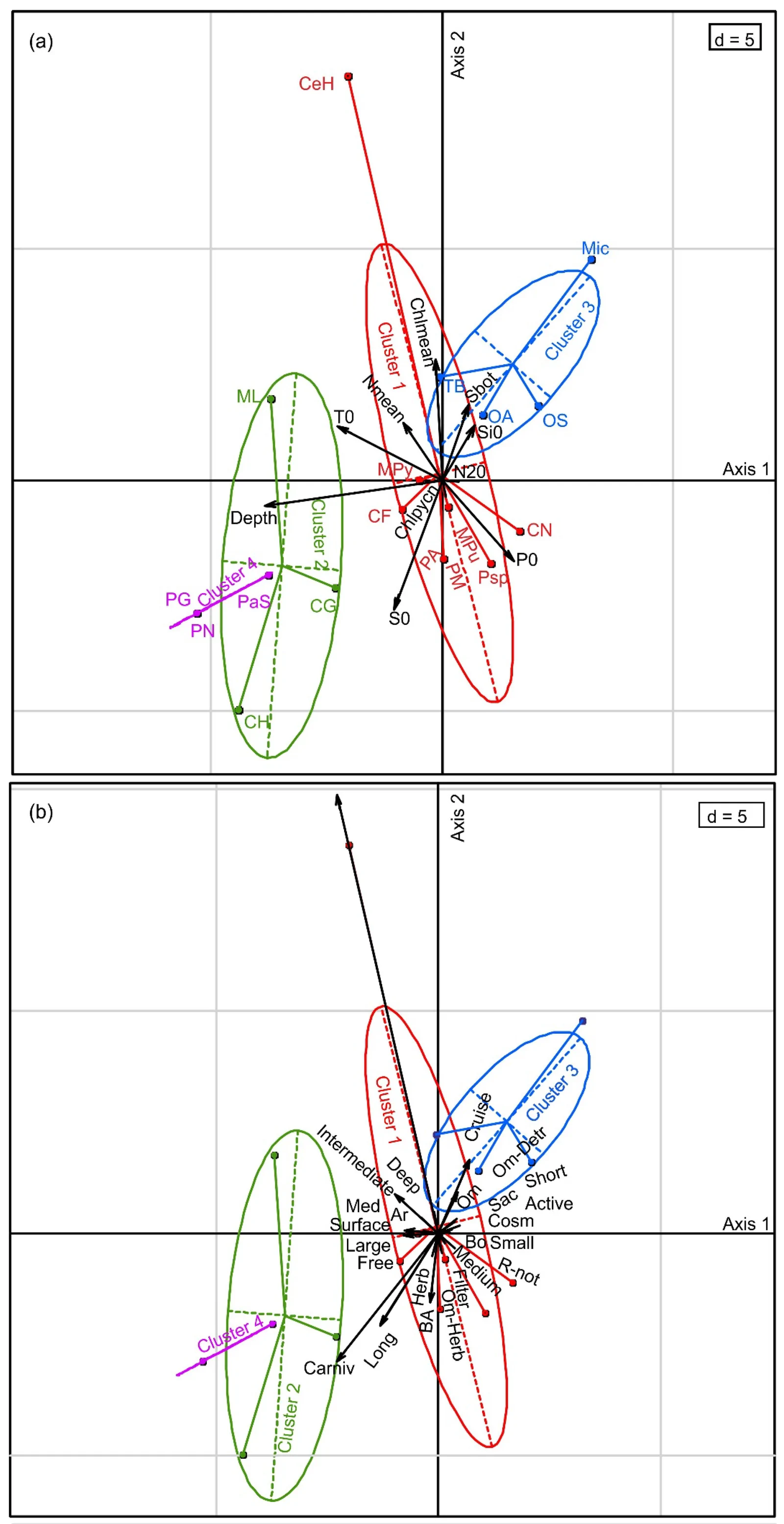
RLQ analysis ordination (axes 1 and 2) illustrating (**a**) species–environment relationships and (**b**) species–trait relationships for the copepod assemblage, central Barents Sea, summer 2015. Trait abbreviations: size (S—small, M—medium, L—large); trophic mode (Om Herb—omnivore–herbivore, Om—omnivore, Om Detr—omnivore–detritivore, Carniv—carnivore); feeding mode (Active ambush, Filter, Cruising, Mixed); reproductive strategy (Free—free spawning, Sac—sac spawning, R not—non reproductive); life span (Short, Md—medium, Long); preferred habitat (Surface, Intermediate, Deep); geographic distribution (Bo—boreal, Ar—Arctic, BA—boreal–Arctic, Cosm—cosmopolitan).

Copepod taxa grouped into clusters based on their traits demonstrated different associations with environmental variables (Figure 9a). Most taxa of group 1 were positively correlated with Axis 1 and negatively correlated with Axis 2. Their abundances were positively associated with surface phosphate, salinity, nitrite, and negatively with mean Chl-a, whereas *C. hamatus* displayed a positive correlation with mean Chl-a and nitrate concentration (Figure 9a). Copepods of groups 2 and 4 were plotted closely on the left side of the diagram and were strongly correlated with sampling depth (Figure 9a). Group 3 was located in the upper right side of the plot and showed positive correlations with mean Chl-a, bottom salinity, and surface silicate (Figure 9a). Trait characteristics of copepods differed along the main environmental gradients (Figure 9a,b; Table 4 and Table 5). Large, carnivorous, long-lived, and intermediate-depth copepods were positively associated with sampling depth, surface temperature, salinity, and mean Chl-a (Figure 9a,b; Table 5). Small, omnivorous, omnivore–detritivore, short-lived, sac-spawning, and mixed-feeding copepods showed positive correlations with mean nitrate and Chl-a concentrations, surface silicate, and bottom salinity (Figure 9a,b). Medium-sized, filter-feeding, herbivorous, and non-reproducing taxa were positively correlated with surface salinity and phosphate concentration (Figure 9a,b).

The fourth-corner analysis was applied to test the significance of bivariate trait–environment associations (9999 permutations). The analysis revealed significant correlations for the following pairs: large vs. depth (*p* = 0.031), omnivore–herbivore vs. mean Chl-a (*p* = 0.041), omnivore vs. mean Chl-a (*p* = 0.042), cruise-feeding vs. surface temperature (*p* = 0.036), cruise-feeding vs. surface phosphate (*p* = 0.005), cruise-feeding vs. mean nitrate (*p* = 0.029), mixed-feeding vs. mean Chl-a (*p* = 0.024), long-lived vs. bottom salinity (*p* = 0.039), intermediate (habitat) vs. bottom salinity (*p* = 0.029), Arctic (zoogeographic group) vs. depth (*p* = 0.013), Arctic vs. surface temperature (*p* = 0.043), and cosmopolitan vs. depth (*p* = 0.004). Sampling depth was found to be the most important factor affecting copepod traits in the study area, especially body size and zoogeographic patterns (*p* < 0.05). RDA showed that diversity and functional diversity metrics were controlled by sampling depth (Table 6), which accounted for 16% (diversity) and 11% (functional diversity) of the total variation. All sets of environmental variables explained 34% and 27% of the total variability in diversity and functional diversity, respectively. The sensitivity analysis demonstrated that the core trait–environment relationships were highly robust to the exclusion of taxa that may belong to more than one functional category (*Pseudocalanus* spp. and copepod nauplii). The overall structure of the associations remained largely unchanged following the reduction in the dataset, as evidenced by a high RV coefficient of 0.834 between the full and reduced fourth-corner matrices (*p* = 0.001, based on 999 permutations). This strong structural concordance confirms that the main ecological gradients and trait–environment linkages are stable, and the overall community assembly patterns are not disproportionately driven by the presence of the excluded species.

## 4. Discussion

### 4.1. Environmental Conditions

Spatial distributions of the main water properties reflected the influence of the major currents in the Barents Sea. The western sub-region had lower Chl-a and lower temperatures, which may be explained by the influence of colder currents flowing from the Arctic Ocean into the Barents Sea, whereas the eastern part was mainly affected by warmer waters extending into the Barents Sea as an Atlantic inflow [4]. Salinity showed low spatial variability and was close to true oceanic values, suggesting a strong impact of Atlantic Water [6,7,9].

Dissolved oxygen concentration was not directly related to phytoplankton abundance and demonstrated an opposite pattern compared with Chl-a. One might expect increased dissolved oxygen in regions with high Chl-a due to the photosynthetic activity of marine microalgae [3,77]. We suggest that both biotic and abiotic processes were responsible for the distribution of dissolved oxygen in the eastern part of the study area. First, lower concentrations might be related to higher abundances of zooplankton in the eastern sub-region [24,78,79]. Second, wind intensity in the east was lower than in the west during our cruise, which led to decreased oxygen intrusion into the surface layer from the atmosphere [80].

The spatial distribution of inorganic nutrients (nitrate and phosphate) was characterized by reduced concentrations in the eastern sub-region. This depletion is attributable to intensified phytoplankton biomass, as evidenced by the elevated Chl-a levels. The northern region is characterized by relatively high summer bacterioplankton abundance and biomass [81] that may explain the enhanced amounts of nitrite. Silicate had two peaks in the northern and southern parts of the study area. Two factors are considered to be the most important in controlling silicate distribution: the distribution of diatoms, which consume significant amounts of silicate, and the position of the pycnocline in oceanic waters [29,82].

Comparisons of the hydrological parameters recorded in summer 2015 with observations made in previous years [6,80] suggest a warming pattern in the central Barents Sea during our research period. This finding is consistent with recent climatic observations in the Arctic, which indicate a warming trend since the early 21st century [5,8,9,10].

### 4.2. Copepod Abundance and Biomass

The copepod assemblage in the central Barents Sea represents a mixture of Arctic, boreal, and cosmopolitan taxa [1,5,18,19,24,25,36,83]. All taxa recorded in this study are typical members of zooplankton inhabiting frontal zones in the Barents Sea [1,3,19,36]. Previous investigations have highlighted small copepods (e.g., *Oithona*, *Triconia*, *Microcalanus*, *Pseudocalanus* spp.) as dominating in terms of total zooplankton abundance during the summer periods [19,36,78,79,84,85,86,87]. Conversely, larger taxa (*Calanus* spp., *Metridia*, *Paraeuchaeta* spp.) prevail in terms of total zooplankton biomass. Moreover, *C. finmarchicus* and *C. glacialis* comprise up to 80–90% of the copepod biomass in summer and autumn in the Barents Sea Water mass [20,21,30,31].

Absolute values of copepod abundance and biomass in this study were comparable to estimates obtained in other warm years, but higher than those observed in cold years [19,20,21,36,80]. A positive relationship between warmer climatic conditions and zooplankton abundance and biomass has been noted in several previous studies [2,22,24,78,79,87]. Rising temperature promotes boreal and cosmopolitan taxa by accelerating growth and reproduction [15,84].

### 4.3. Functional Groups of Copepods

Analysis of our dataset allowed us to delineate four distinct copepod functional groups in the central Barents Sea. We used several traits to separate copepod species, but size, feeding mode, trophic preference, and life cycle duration were the most important characteristics. Distribution patterns and zoogeographic features were of minor importance because most taxa are capable of vertical migration. Medium-sized and small omnivore–herbivore species with a current-feeding trophic mode, short life cycles, and occurrence in the upper water layer constituted group 1. *Calanus finmarchicus*, Copepoda nauplii, *Microcalanus* spp., and *Pseudocalanus* spp. are typical taxa inhabiting all regions of the Barents Sea but are reaching maximum abundances in warmer waters [19,20,21,26,32,33,34,35,36]. *Centropages hamatus* is a neritic copepod inhabiting mainly the southern regions [25,26,83]. Abundances of these taxa are strongly linked to the phytoplankton cycle in the Barents Sea [1,84,88]. Therefore, they peak during or after the main phytoplankton bloom, in April–May in the southern regions and in July–August in the central regions [16,31]. Consequently, they play a significant role in utilizing primary production and are deeply involved in carbon cycling in Atlantic Water and Murmansk Coastal Water (*C. finmarchicus*, *C. hamatus*, and *Microcalanus* spp.), and in Barents Sea Water (*Pseudocalanus* spp.) [19,20,21,34,35,36]. Dominance of current-feeding copepods has also been found in the Arctic Basin and south of the Polar Front in July–August [38].

Larger *Calanus* copepods and *M. longa* were combined in group 2. These species occur in great numbers in colder waters [2,3,27,30,61]. The two *Calanus* species inhabit the northern regions and are considered indicators of the Arctic domain in the Barents Sea, whereas *M. longa* has a broader distribution but prefers intermediate and deep waters [18,19,20,21,28,33,36]. They regulate carbon cycling in ice-covered regions, serving as a link between phytoplankton and higher trophic levels. The total abundance of this group was high in the north, reflecting its association with cold waters. A second region with enhanced abundance of group 2 was the south-east, where high densities of *M. longa* were found. Similar results have been documented for the northern and northwestern Barents Sea, where large copepod species were associated with cold waters [18,38].

The third functional group encompassed the small cyclopoid copepods of the genus *Oithona* together with the harpacticoid *Microsetella norvegica* and the poecilostomatoid *Triconia borealis*. These short-lived species can be found in all regions of the Barents Sea [61]. They can attain high numbers throughout the year and exploit a wide food spectrum, ranging from phytoplankton and microzooplankton to detritus and marine microbes [17,29,82,89]. Most of these taxa show a preference for surface and subsurface layers and for relatively warm waters. Durazzano et al. [38] noted a similar pattern for small copepods in the northwestern Barents Sea. Therefore, they play a crucial role as mediators among mesozooplankton, phytoplankton, and microbial assemblages [90]. By utilizing detritus, they are among the main zooplankters involved in biogeochemical cycling during the winter, when phytoplankton biomass is extremely low [23,91].

Large, cruise-feeding *Paraeuchaeta* species with long life cycles were combined into group 4. As carnivores, they are important consumers of mesozooplankton biomass [92]. A characteristic feature of *P. norvegica* and *P. glacialis* is their preference for deep waters [18,93,94]. Similar to our findings, cruise-feeding copepods have been reported as prevailing in the intermediate and deep waters of the northwestern Barents Sea [38].

### 4.4. Taxonomic Diversity and Functional Diversity

Copepod taxonomic diversity and functional diversity showed different degrees of variability in the central Barents Sea. The Shannon diversity index fluctuated widely, with a maximum in the northern sub-region. Thus, this area represented a hotspot of copepod diversity in our study. It is worth noting that the total abundance of functional groups 1, 2, and 4 was also high in this region, suggesting a complex ecological structure of the pelagic ecosystem and a higher number of ecological niches available for copepods. This sub-region, characterized by variable water depth, provides habitats for surface, intermediate, and deep-water taxa [61].

In addition, the area is located near the frontal zones separating Barents Sea Water and Novaya Zemlya Coastal Water, which creates ecological niches for taxa with different thermal preferences [5]. Frontal zones are typically associated with enhanced primary productivity, which promotes the growth and development of herbivorous and omnivore-herbivorous copepods that numerically dominate in the central Barents Sea [19,20,21,36].

In contrast, the number of taxa and evenness showed low variability within the study area, suggesting the dominance of approximately 3–5 taxa without extreme peaks of a single species. This pattern is expected because zooplankton in Barents Sea Water consist of a mixture of boreal, boreal–Arctic, Arctic, and cosmopolitan species [19,61,87]. Their co-existence is strongly associated with the regional current system and the origin of Barents Sea Water, which is formed from Atlantic Water by cooling due to the intrusion of waters from the northern Barents Sea [3,77]. Species dominance (the inverse Simpson index) was also low, supporting the conclusion that several dominant taxa are present but no single taxon numerically overwhelms the copepod assemblage.

The diversity metrics observed in this research are in good agreement with other investigations conducted in the Barents Sea during the summer [19,36,95]. Typically, 3–4 taxa dominate in different water masses. For instance, Atlantic Water is characterized by high relative abundances of *O. similis*, *C. finmarchicus* and *Pseudocalanus* spp., whereas *C. glacialis*, Copepoda nauplii and *O. similis* prevail in Arctic Water in June–July [1,20,21,22,28,61].

Functional dispersion, functional mean pairwise distance, and functional mean nearest neighbor distance were relatively stable across the study area, indicating low variability in taxa and in distances between taxa in trait space. This pattern can be explained by the location of most stations within a single dominant water mass, where fluctuations in environmental filters were relatively weak [5,6,77]. Functional evenness showed higher spatial variability, with maximum values in the south-eastern and western parts of the study area. In these sub-regions, species abundances appear to be more regularly distributed within functional trait space. Consequently, we propose that copepod assemblages there may have higher ecological resilience to environmental disturbances, consistent with findings from other oceanic regions, including the South Atlantic Ocean [49], the northwestern Pacific Ocean [50] and the Southern Ocean [46]. Regions with lower functional evenness may be more vulnerable to the loss of species with rare functional traits and may exhibit lower ecosystem resilience [37,96]. Higher functional evenness has been associated with an even distribution of zooplankton with diverse traits in functional space, suggesting complementary resource use, as reported for the North Pacific Subtropical Gyre [50]. It is important to note that ecosystem resilience was not directly measured in this study. The proposed link between functional evenness and resilience is therefore an implication drawn from other similar studies rather than a finding supported by our data. Higher functional evenness may suggest a more equitable distribution of traits and potentially greater capacity to buffer environmental change, but this interpretation requires subsequent testing. Future studies should combine functional diversity metrics with direct measures of ecosystem stability, such as resistance and recovery rates, to evaluate this hypothesis.

Functional richness corresponded well with the number of taxa and was highest in the south-eastern sub-region. This suggests high representation of the main functional groups during the summer of 2015 and higher productivity of pelagic communities there. Functional divergence was relatively stable, indicating low variation in niche overlap and competition within the copepod assemblage [96], which is consistent with low variability in environmental conditions. The south-western region exhibited relatively high functional divergence, suggesting that copepods there may use food resources more efficiently, with greater niche differentiation and reduced competition for food [69]. In this study, higher functional richness indicated that the copepod assemblage occupied a larger volume of the functional trait space, reflecting a broader representation of the main functional groups. Because functional richness was calculated from the range of trait values present, higher values mean that the assemblage included taxa with more divergent combinations of size, feeding mode, trophic mode, and life-history traits. This interpretation is therefore based on the trait-space definition of functional richness rather than on species number alone.

Functional originality is a metric indicating taxa with unique ecological roles. We identified higher functional originality in the northern region, most likely associated with the presence of uncommon *Paraeuchaeta* species, which were assigned to functional group 4. Functional specialization measures the degree of adaptation of a species to a narrow range of environmental conditions and reflects its functional role in the pelagic realm. Our study found no clear spatial variations in functional specialization, suggesting that copepods shared broadly similar environmental preferences and were well adapted to the water properties encountered in the central Barents Sea [19,20,21,36]. Thus, we confirmed our hypothesis that the copepod assemblage and the functional traits of particular taxa were broadly similar within the study area.

### 4.5. Associations of Copepod Traits, Diversity, and Functional Diversity with Environmental Variables

Our RLQ analysis showed a strong correlation between copepod body length and sampling depth, with small and medium-sized taxa inhabiting surface and intermediate layers and large taxa occurring mainly in deeper waters. Temperature had a similar but statistically non-significant effect on size structure (*p* > 0.05). As already noted, temperature variability was low in the study region, and its influence on body-size fluctuations was therefore weaker than that of other factors. Sampling depth also explained spatial variations in the zoogeographic structure of copepods in the central Barents Sea. Arctic and boreal–Arctic taxa were associated with shallower depths, whereas boreal taxa occurred predominantly in deeper waters. This pattern is likely linked to the regional current system [4,19,36]. Arctic taxa inhabit mainly surface layers influenced by cold currents in the northern and partly central regions [3,5]. Boreal taxa (e.g., *C. finmarchicus* and *O. atlantica*) show close associations with Atlantic Waters located in intermediate layers [16,84].

The fourth-corner analysis further clarified associations between functional traits and environmental factors. Sampling depth was identified as a key variable affecting the abundance of large and cosmopolitan taxa. The correlation of cosmopolitan taxa with depth can be explained by their preference for upper layers. For example, small cosmopolitan copepods such as *O. similis*, *M. norvegica* and Copepoda nauplii typically colonize surface and subsurface layers where food resources are abundant during summer [24,25,33,61,85,87,97]. A previous investigation in the northwestern Barents Sea also revealed that a depth gradient explained a significant proportion of variation in traits of copepod functional groups based on RDA results [38].

Surface water temperature was an important factor for the distribution of cruise-feeding copepods and Arctic taxa in our study. Both trait groups were negatively correlated with this variable, indicating higher abundances of these copepods in colder waters, both in deep layers (e.g., *M. longa*, *T. borealis* and *Paraeuchaeta* spp.) and in northern regions influenced by cold currents (e.g., *C. glacialis*, *C. hyperboreus*, *M. pygmaeus*). Similar findings have been reported in the northwestern Barents Sea, where large filter-feeding copepods were associated with highly stratified, colder waters, whereas particle-feeding taxa were correlated with warmer waters [38].

Bottom salinity explained the spatial patterns of long-lived and intermediate-depth taxa, showing a positive association with higher salinity. This result is expected because higher salinities are encountered in Atlantic Waters flowing at the intermediate and deep-water layers [6] in the central Barents Sea.

Mean nitrate concentration was positively related to the abundance of cruise-feeding copepods, whereas surface phosphate concentration was negatively correlated with this group. This pattern primarily reflects the association of cruise-feeding copepods with relatively nutrient-rich waters in deeper and intermediate layers [77]. Mean Chl-a concentrations were positively related to the abundances of mixed-feeding, omnivorous, and omnivore–detritivore copepods, which together constituted the majority of copepod abundance in our study. In contrast, the omnivore–herbivore taxa exhibited an inverse relationship with Chl-a. Microalgae represents a substantial component of the diet of the dominant copepod taxa in the Barents Sea, particularly in the spring [1,3,16,88]. Consequently, high abundances of these functional groups would be expected in regions characterized by elevated Chl-a concentrations.

RDA revealed that a set of diversity and functional diversity metrics was strongly associated with sampling depth. While this highlights depth as the strongest measured predictor in our dataset, we interpret this pattern with caution. In regions with weak horizontal environmental gradients, depth often acts as a proxy for the vertical extent of the water column rather than as an independent ecological driver.

An important caveat of our research is that zooplankton samples were collected by vertical hauls from near the bottom to the surface. Consequently, the vertical extent of the sampled water column varied with station depth: deeper stations encompassed a larger range of potential habitats and had the capacity to sample deep-water taxa absent from shallower stations. Therefore, observed relationships between station depth and habitat-related traits (such as the association of large or Arctic taxa with greater depth) may partly reflect sampling design and habitat availability rather than a direct, depth-specific ecological effect.

Accordingly, our initial hypothesis that trait characteristics and diversity indices would be broadly controlled by multiple independent environmental factors was not fully supported, largely due to the absence of clear horizontal gradients. The observed depth-related patterns remain valid, but depth in this context should be consistently described as an integrated descriptor of vertical habitat extent, water-mass structure, and sampling coverage. Future studies should employ stratified vertical sampling to disentangle true depth preferences from the effects of habitat availability.

## 5. Conclusions

Based on the principal characteristics of copepods inhabiting the central Barents Sea during summer, four functional groups were delineated. Among the measured environmental variables, sampling depth was the strongest predictor of spatial variation in copepod diversity, functional diversity, and several functional traits. However, depth explained only a limited proportion of the total variation, and its effect should be interpreted cautiously because vertical hauls integrated the water column from near the bottom to the surface, so deeper stations sampled a larger vertical habitat range and may have included deep-water taxa that were absent at shallower stations. Other environmental variables had weaker effects and were associated mainly with particular trait modalities rather than with overall assemblage structure. Regional currents and circulation patterns may mediate the distribution of functional groups, but environmental gradients within the study area were narrow, and most stations were located within a single water-mass type. The RLQ and fourth-corner analyses identified trait–environment associations, particularly involving depth, temperature, chlorophyll *a*, and nutrients, but these relationships were correlative and should be treated as exploratory. Taxonomic and functional diversity metrics both varied spatially, but their direct association was not explicitly tested in this study; we therefore do not infer a close coupling between them or use them as direct evidence of ecological resilience. Overall functional originality was low, which is consistent with sampling within one predominant water mass. This study provides baseline data on copepod functional traits in the central Barents Sea and can serve as a reference for comparisons with assemblages from other Arctic regions. Continued monitoring of zooplankton functional diversity will enhance the understanding of pelagic ecosystem functioning under ongoing global climate change. Future work should expand spatial coverage, use stratified vertical sampling, and include additional seasons to elucidate the mechanisms controlling the temporal and spatial dynamics of zooplankton traits in Arctic marine communities.

## Figures and Tables

**Table 1:** Traits of copepods used to separate functional groups in the central Barents Sea in summer 2015.

Trait	1	2	3	4
Size	Small (<1 mm)	Medium (1–2 mm)	Large (>2 mm)	
Trophic mode	Omnivore–herbivore	Omnivore	Omnivore–detritovore	Carnivore
Feeding strategy	Active ambush	Filter	Cruising	Mixed
Reproduction mode	Free spawning	Sac spawning	Not reproducing	
Life span	Short (<1 year)	Medium (1–2 years)	Long (>2 years)	
Habitat	Surface (0–50 m)	Intermediate (50–200 m)	Deep (200 m–bottom)	
Zoogeography	Boreal	Arctic	Boreal–Arctic	Cosmopolite

**Table 2:** Environmental parameters measured in the central Barents Sea, summer 2015: variable name, units, range, and mean ± SD.

Parameter.	Min–Max	Mean ± SD
Surface temperature (°C)	3.2–6.5	4.9 ± 1.0
Bottom temperature (°C)	−0.4–2.1	0.6 ± 0.6
Mean temperature (°C)	0.7–3.7	1.8 ± 0.8
Surface salinity	34.76–35.68	34.97 ± 0.21
Bottom salinity	34.8–35.02	34.99 ± 0.04
Mean salinity	34.88–34.98	34.95 ± 0.02
Surface Chl-a (mg m^−3^)	0.14–1.67	0.59 ± 0.41
Bottom Chl-a (mg m^−3^)	0.01–0.62	0.24 ± 0.13
Mean Chl-a (mg m^−3^)	0.2–0.72	0.43 ± 0.13
Surface phosphate (μg L^−1^)	6–73	27 ± 14
Bottom phosphate (μg L^−1^)	63–122	87 ± 15
Mean phosphate (μg L^−1^)	32–86	56 ± 11
Surface dissolved oxygen (mg L^−1^)	11–13	12 ± 0
Bottom dissolved oxygen (mg L^−1^)	11–12	11 ± 0
Mean dissolved oxygen (mg L^−1^)	11–12	12 ± 0
Surface nitrite (μg L^−1^)	0–10	3 ± 2
Bottom nitrite (μg L^−1^)	0–9	4 ± 2
Mean nitrite (μg L^−1^)	1–8	4 ± 2
Surface nitrate (μg L^−1^)	15–188	42 ± 26
Bottom nitrate (μg L^−1^)	14–324	218 ± 54
Mean nitrate (μg L^−1^)	77–172	130 ± 19
Surface silicate (μg L^−1^)	0–297	60 ± 63
Bottom silicate (μg L^−1^)	0–318	164 ± 88
Mean silicate (μg L^−1^)	13–187	100 ± 41

**Table 3:** Mesozooplankton composition, mean abundance of taxa (individuals m^−3^), total abundance (individuals m^−3^), integrated abundance (10^3^ individuals m^−2^), evenness (E), Shannon index (H’), total biomass (mg dry mass m^−3^), integrated biomass (g dry mass m^−2^) in the central Barents Sea, summer 2015.

	Abundance		Biomass	
Taxon	Min–Max	Mean ± SD	Min–Max	Mean ± SD
*Calanus finmarchicus*	58–270	125 ± 47	3.5–21.4	11.4 ± 4.5
*Calanus glacialis*	12–125	48 ± 25	1.5–44.2	15.7 ± 10
*Calanus hyperboreus*	0–49	13 ± 10	0–19.9	4.0 ± 4.4
*Centropages hamatus*	0–11	0.5 ± 2.2	0–0.1	<0.1
Copepoda nauplii	21–212	93 ± 42	0–0.2	0.1 ± 0.1
*Metridia longa*	12–190	103 ± 44	0.6–13	6.3 ± 3.3
*Microcalanus pusillus*	1–8	3.2 ± 1.5	0–0.003	<0.1
*Microcalanus pygmaeus*	5–38	21 ± 8	0–0.1	<0.1
*Microsetella norvegica*	0–2	0.3 ± 0.5	0–0.0002	<0.1
*Oithona atlantica*	0–30	10 ± 7	0–0.003	<0.1
*Oithona similis*	102–431	211 ± 76	0.1–0.4	0.2 ± 0.1
*Triconia borealis*	0–3	0.6 ± 0.8	0–0.0001	<0.1
*Paraeuchaeta* spp. I–IV	0–0.4	0 ± 0.1	0–0.1	<0.1
*Paraeuchaeta glacialis*	0–0.2	<0.1	0–0.6	<0.1
*Paraeuchaeta norvegica*	0–0.1	<0.1	0–0.2	<0.1
*Pseudocalanus* spp. I–IV	21–154	55 ± 27	0.1–0.6	0.2 ± 0.1
*Pseudocalanus minutus*	15–70	34 ± 15	0.2–0.9	0.4 ± 0.2
*Pseudocalanus acuspes*	4–37	15 ± 9	0–0.3	0.1 ± 0.1

**Table 4:** Correlations of environmental variables with the first two RLQ axes (axis 1 and axis 2) for the central Barents Sea, summer 2015.

Variable	Axis 1	Axis 2
Sampling depth	−0.90	0.08
Surface temperature	−0.67	0.60
Surface salinity	−0.17	−0.71
Bottom salinity	0.10	0.63
Mean Chl-a	−0.15	0.79
Surface phosphate	0.64	−0.34
Surface nitrite	0.33	0.17
Mean nitrate	−0.23	0.42
Surface silicate	0.10	0.35

**Table 5:** Correlations of species traits with the first two RLQ axes (axis 1 and axis 2) for the central Barents Sea, summer 2015.

Trait	Axis 1	Axis 2
Small	0.78	0.24
Medium	−0.29	−0.56
Large	−1.58	0.02
Carnivore	−2.05	−1.43
Omnivore	0.08	0.93
Omnivore–detritovore	1.53	2.89
Omnivore–herbivore	−0.07	−0.84
Active ambush	0.96	0.97
Cruising	−1.74	1.06
Filter	−0.08	−0.81
Mixed	−0.96	5.29
Free spawning	−0.87	−0.26
Not reproduced	0.68	−0.83
Sac spawning	0.96	0.97
Short life cycle	0.70	0.10
Medium life cycle	−1.03	−0.02
Long life cycle	−2.09	−3.01
Habitat Deep	1.24	2.56
Habitat Intermediate	−0.95	0.30
Habitat Surface	0.44	−0.77
Arctic	−1.43	0.02
Boreal–Arctic	0.26	−1.01
Boreal	−0.34	−0.28
Cosmopolitan	0.93	0.47

**Table 6:** Redundancy analysis (RDA) results: influence of environmental variables on taxonomic diversity and functional diversity metrics of copepods in the central Barents Sea, summer 2015. Table reports canonical coefficients, F statistics and significance (permutation tests). Bold font indicates significant factor.

			Monte-Carlo Test	
Factor	Axis1	Axis 2	F	*p*
	Diversity			
**Sampling depth**	**0.40**	**−0.10**	**5.41**	**<0.02**
Surface temperature	0.13	0.01	0.97	0.35
Surface salinity	−0.15	−0.05	0.69	0.50
Bottom salinity	0.08	0.08	0.04	0.99
Mean Chl-a	0.15	0.23	0.18	0.84
Surface phosphate	−0.27	0.01	0.72	0.46
Surface nitrite	−0.01	0.11	0.61	0.54
Mean nitrate	0.20	−0.22	1.51	0.22
Surface silicate	0.04	0.24	0.27	0.78
	Functional diversity			
**Sampling depth**	**−0.53**	**0.07**	**7.92**	**<0.03**
Surface temperature	−0.33	−0.06	1.22	0.32
Surface salinity	−0.24	−0.09	1.97	0.15
Bottom salinity	0.10	−0.02	0.04	0.98
Mean Chl-a	0.00	0.15	1.61	0.22
Surface phosphate	0.13	−0.07	0.27	0.77
Surface nitrite	−0.09	0.08	1.79	0.18
Mean nitrate	−0.20	0.18	0.95	0.41
Surface silicate	0.14	0.07	0.90	0.38

## Data Availability

The data supporting this study’s findings are available from the corresponding authors upon reasonable request.

## Supplementary Materials

The following supporting information can be downloaded at: https://www.mdpi.com/article/10.3390/ani16213405/s1. Table S1: Sampling stations performed in the central Barents Sea, summer 2015: station ID, sampling date, sampling time, geographic coordinates, and sampling layer (m). Table S2: Functional traits for taxa found in the central Barents Sea, summer 2015. References [98,99,100,101,102,103,104,105,106,107] are cited in Supplementary Materials.
