# Supplementary material for: Functional Groups of Summer Copepod Assemblage in the Central Barents Sea
Source: Animals (Basel). 2026 Oct 28;16(21):3405. doi: 10.3390/ani16213405 (PMC13762151; doi:10.3390/ani16213405)
Supplement: Supplementary file 1 [file animals-16-03405-s001.zip › animals-4578423-supplementary.pdf]

Functional groups of summer copepod assemblage in the central Barents Sea

Vladimir G. Dvoretsky and Alexander G. Dvoretsky

Table S1. Sampling stations performed in the central Barents Sea, summer 2015: station ID, sampling date, sampling time, geographic coordinates, and sampling layer (m).

ID	Date	Time	Latitude	Longitude	Sampling layer
1	11-Jul-15	16:45	73°49´	46°29´	0–270
2	11-Jul-15	05:06	73°55´	46°58´	0–240
3	10-Jul-15	14:55	74°01´	47°23´	0–270
4	10-Jul-15	06:20	74°07´	47°11´	0–250
5	10-Jul-15	21:52	74°02´	46°43´	0–240
6	11-Jul-15	10:23	73°56´	46°09´	0–270
7	9-Jul-15	18:56	74°22´	46°01´	0–270
8	9-Jul-15	13:20	74°29´	46°11´	0–250
9	4-Jul-15	17:50	74°45´	47°45´	0–230
10	4-Jul-15	10:02	74°49´	48°34´	0–240
11	4-Jul-15	03:55	74°57´	48°12´	0–230
12	4-Jul-15	23:45	74°51´	47°24´	0–225
13	5-Jul-15	07:34	74°47´	46°55´	0–200
14	7-Jul-15	14:40	74°44´	46°35´	0–190
15	8-Jul-15	21:17	74°32´	45°43´	0–300
16	8-Jul-15	12:55	74°46´	44°57´	0–280
17	8-Jul-15	05:50	74°46´	45°27´	0–280
18	7-Jul-15	23:42	74°52´	46°13´	0–245
19	3-Jul-15	09:15	74°59´	47°00´	0–230
20	3-Jul-15	19:17	75°01´	47°38´	0–250
21	30-Jun-15	08:45	75°35´	43°27´	0–300
22	30-Jun-15	18:01	75°35´	44°07´	0–300
23	1-Jul-15	01:30	75°35´	44°53´	0–290
24	30-Jun-15	00:45	75°22´	43°27´	0–300
25	2-Jul-15	01:10	75°22´	43°57´	0–300
26	1-Jul-15	18:06	75°22´	44°27´	0–290
27	1-Jul-15	08:05	75°22´	44°53´	0–290
28	29-Jun-15	17:55	75°08´	43°27´	0–300
29	2-Jul-15	08:35	75°08´	44°07´	0–300
30	2-Jul-15	18:02	75°08´	44°53´	0–270
31	26-Jun-15	18:21	74°23´	42°19´	0–230
32	26-Jun-15	22:15	74°28´	41°56´	0–200
33	23-Jun-15	14:00	74°34´	41°25´	0–200
34	24-Jun-15	12:52	74°38´	40°58´	0–170
35	23-Jun-15	19:01	74°40´	41°41´	0–250
36	27-Jun-15	04:07	74°34´	42°13´	0–190
37	27-Jun-15	10:10	74°35´	42°57´	0–280
38	27-Jun-15	17:32	74°44´	42°14´	0–180
39	24-Jun-15	07:01	74°52´	41°32´	0–170
40	29-Jun-15	01:35	74°53´	42°23´	0–200
41	27-Jun-15	23:15	74°49´	42°46´	0–245
42	28-Jun-15	06:43	74°49´	43°34´	0–310
43	28-Jun-15	14:10	74°55´	43°04´	0–270
44	29-Jun-15	08:25	75°00´	42°37´	0–220
45	24-Jun-15	01:20	75°06´	42°10´	0–200

Table S2. Functional traits for taxa found in the central Barents Sea, summer 2015.

Taxon	Size	Trophic mode	Feeding strategy	Reproduction mode	Life span	Habitat	Zoogeography	Reference
Calanus finmarchicus	Medium	Omnivore-Herbivore	Filter feeding	Free spawning	Medium (1-2 years)	Surface (0-50 m)/Intermediate (50-200 m)	Boreal	Jaschnov, 1948; Brodsky, 1967; Brodskiy et al., 1983; Mumm, 1991; Richter, 1994; Mauchline, 1998; Falk-Petersen et al., 2007; Dvoretsky and Dvoretsky, 2010; Pomerleau et al., 2015; Benedetti et al., 2016; Razouls et al., 2025
Calanus glacialis	Large	Omnivore-Herbivore	Filter feeding	Free spawning	Medium (1-2 years)	Surface (0-50 m)	Arctic	Jaschnov, 1948; Brodsky, 1967; Brodskiy et al., 1983; Mumm, 1991; Richter, 1994; Mauchline, 1998; Falk-Petersen et al., 2007; Dvoretsky and Dvoretsky, 2010; Pomerleau et al., 2015; Benedetti et al., 2016; Razouls et al., 2025
Calanus hyperboreus	Large	Omnivore-Herbivore	Filter feeding	Free spawning	Long (>2 years)	Intermediate (50-200 m)	Arctic	Jaschnov, 1948; Brodsky, 1967; Brodskiy et al., 1983; Mumm, 1991; Richter, 1994; Mauchline, 1998; Falk-Petersen et al., 2007; Dvoretsky and Dvoretsky, 2010; Pomerleau et al., 2015; Benedetti et al., 2016; Razouls et al., 2025
Centropages hamatus	Small	Omnivore-Herbivore	Mixed	Free spawning	Short (<1 year)	Surface (0-50 m)	Boreal-Arctic	Jaschnov, 1948; Brodsky, 1967; Brodskiy et al., 1983; Mauchline, 1998; Dvoretsky and Dvoretsky, 2010; Pomerleau et al., 2015; Benedetti et al., 2016; Becker et al., 2021; Razouls et al., 2025
Copepoda nauplii	Small	Omnivore-Herbivore	Filter feeding	Not reproducing	Short (<1 year)	Surface (0-50 m)	Cosmopolite	Mauchline, 1998; Dvoretsky and Dvoretsky, 2010; Barth-Jensen et al., 2020; Barth-Jensen et al., 2022
Metridia longa	Large	Omnivore	Cruisying	Free spawning	Medium (1-2 years)	Intermediate (50-200 m)	Arctic	Jaschnov, 1948; Brodsky, 1967; Haq, 1967; Brodskiy et al., 1983; Mumm, 1991; Richter, 1994; Mauchline, 1998; Dvoretsky and Dvoretsky, 2010
Microcalanus pusillus	Small	Omnivore	Filter feeding	Free spawning	Short (<1 year)	Surface (0-50 m)	Boreal-Arctic	Jaschnov, 1948; Brodsky, 1967; Brodskiy et al., 1983; Mumm, 1991; Richter, 1994; Mauchline, 1998; Dvoretsky and Dvoretsky, 2010; Barth-Jensen et al., 2020; Barth-Jensen et al., 2022
Microcalanus pygmaeus	Small	Omnivore	Filter feeding	Free spawning	Short (<1 year)	Surface (0-50 m)	Arctic	Jaschnov, 1948; Brodsky, 1967; Brodskiy et al., 1983; Mumm, 1991; Richter, 1994; Mauchline, 1998; Dvoretsky and Dvoretsky, 2010; Pomerleau et al., 2015; Benedetti et al., 2016; Razouls et al., 2025
Microsetella norvegica	Small	Omnivore-Detritovore	Cruisying	Sac spawning	Short (<1 year)	Surface (0-50 m)	Cosmopolite	Jaschnov, 1948; Diaz and Evans, 1983; Dvoretsky and Dvoretsky, 2010; Barth-Jensen et al., 2020; Barth-Jensen et al., 2022
Oithona atlantica	Small	Omnivore	Ambush	Sac spawning	Short (<1 year)	Intermediate (50-200 m)	Boreal	Jaschnov, 1948; Dvoretsky and Dvoretsky, 2010; Benedetti et al., 2016; Razouls et al., 2025
Oithona similis	Small	Omnivore	Ambush	Sac spawning	Short (<1 year)	Surface (0-50 m)/	Cosmopolite	Jaschnov, 1948; Dvoretsky and Dvoretsky, 2010; Barth-Jensen et al., 2020; Barth-Jensen et al., 2022

						Intermediate (50-200 m)		
Triconia borealis	Small	Omnivore	Cruisying	Sac spawning	Short (<1 year)	Intermediate (50-200 m)	Cosmopolite	Jaschnov, 1948; Dvoretsky and Dvoretsky, 2010; Benedetti et al., 2016; Becker et al., 2021; Razouls et al., 2025
Paraeuchaeta spp I-IV	Medium	Carnivore	Cruisying	Not reproducing	Short (<1 year)	Intermediate (50-200 m)	Arctic	Jaschnov, 1948; Brodsky, 1967; Markhaseva, 1996; Mumm, 1991; Richter, 1994; Mauchline, 1998; Brodskiy et al., 1983; Auel, 1999; Dvoretsky and Dvoretsky, 2010; Pomerleau et al., 2015; Benedetti et al., 2016; Razouls et al., 2025
Paraeuhaeta glacialis	Large	Carnivore	Cruisying	Sac spawning	Long (>2 years)	Deep (200-bottom)	Arctic	Jaschnov, 1948; Brodsky, 1967; Markhaseva, 1996; Mumm, 1991; Richter, 1994; Mauchline, 1998; Brodskiy et al., 1983; Auel, 1999; Dvoretsky and Dvoretsky, 2010; Pomerleau et al., 2015; Benedetti et al., 2016; Razouls et al., 2025
Paraeuhaeta norvegica	Large	Carnivore	Cruisying	Sac spawning	Long (>2 years)	Deep (200-bottom)	Arctic	Jaschnov, 1948; Brodsky, 1967; Markhaseva, 1996; Mumm, 1991; Richter, 1994; Mauchline, 1998; Brodskiy et al., 1983; Auel, 1999; Dvoretsky and Dvoretsky, 2010; Pomerleau et al., 2015; Benedetti et al., 2016; Razouls et al., 2025
Pseudocalanus spp. I-IV	Small	Omnivore-Herbivore	Filter feeding	Not reproducing	Short (<1 year)	Surface (0-50 m)	Boreal-Arctic	Jaschnov, 1948; Brodsky, 1967; Mumm, 1991; Richter, 1994; Mauchline, 1998; Brodskiy et al., 1983; Dvoretsky and Dvoretsky, 2010; Pomerleau et al., 2015; Aarbakke et al., 2017; Benedetti et al., 2016; Razouls et al., 2025
Pseudocalanus minutus	Medium	Omnivore-Herbivore	Filter feeding	Sac spawning	Short (<1 year)	Surface (0-50 m)	Boreal-Arctic	Jaschnov, 1948; Brodsky, 1967; Mumm, 1991; Richter, 1994; Mauchline, 1998; Brodskiy et al., 1983; Dvoretsky and Dvoretsky, 2010; Pomerleau et al., 2015; Aarbakke et al., 2017; Benedetti et al., 2016; Razouls et al., 2025
Pseudocalanus acuspes	Medium	Omnivore-Herbivore	Filter feeding	Sac spawning	Short (<1 year)	Surface (0-50 m)	Boreal-Arctic	Jaschnov, 1948; Brodsky, 1967; Mumm, 1991; Richter, 1994; Mauchline, 1998; Brodskiy et al., 1983; Dvoretsky and Dvoretsky, 2010; Pomerleau et al., 2015; Aarbakke et al., 2017; Benedetti et al., 2016; Razouls et al., 2025

Aarbakke, O. N. S.; Fevolden, S. E.; Weydmann, A. Relative Summer Abundances and Distribution of *Pseudocalanus* spp. (Copepoda: Calanoida) Adults in Relation to Environmental Variables in the Nordic Seas and Svalbard Fjords. *Polar Biol.* **2017**, *40*, 51–59.

Auel, H. The Ecology of Arctic Deep-Sea Copepods (Euchaetidae and Aetideidae): Aspects of Their Distribution, Trophodynamics and Effect on the Carbon Flux. *Ber. Polarforsch.* **1999**, *319*, 1–97.

Barth-Jensen, C.; Daase, M.; Ormańczyk, M. R.; Varpe, Ø.; Kwaśniewski, S.; Svensen, C. High Abundances of Small Copepods' Early Developmental Stages and Nauplii Strengthen the Perception of a Non-Dormant Arctic Winter. *Polar Biol.* **2022**, *45*, 675–690.

Barth-Jensen, C.; Koski, M.; Varpe, Ø.; Glad, P.; Wangensteen, O. S.; Præbel, K. Temperature-Dependent Egg Production and Egg Hatching Rates of Small Egg-Carrying and Broadcast-Spawning Copepods *Oithona similis*, *Microsetella norvegica* and *Microcalanus pusillus*. *J. Plankton Res.* **2020**, *42*, 564–580.

Becker, É. C.; Mazzocchi, M. G.; de Macedo-Soares, L. C. P.; Brandão, M. C.; Freire, A. S. Latitudinal Gradient of Copepod Functional Diversity in the South Atlantic Ocean. *Prog. Oceanogr.* **2021**, *199*, 102710.

Benedetti, F.; Gasparini, S.; Ayata, S. D. Identifying Copepod Functional Groups from Species Functional Traits. *J. Plankton Res.* **2016**, *38*, 159–166.

Brodskiy, K. A.; Vishkvartseva, N. V.; Kos, M. S.; Markhaseva, E. L. *Calanoid Copepods from the Seas of the USSR and Adjacent Waters. Part 1*; Nauka Press: Leningrad, Russia, 1983; p. 358 (In Russian).

Brodsky, K. A. *Calanoida of the Far Eastern Seas and Polar Basin of the USSR*; Israel Program for Scientific Translations: Jerusalem, Israel, 1967.

Diaz, W.; Evans, F. The Reproduction and Development of *Microsetella norvegica* (Boeck) (Copepoda, Harpacticoida) in Northumberland Coastal Waters. *Crustaceana* **1983**, 113–130.

Falk-Petersen, S.; Timofeev, S.; Pavlov, V.; Sargent, J. R. Climate Variability and Possible Effects on Arctic Food Chains: The Role of *Calanus*. In *Arctic Alpine Ecosystems and People in a Changing Environment*; Orbok, J. B., Tombre, T., Kallenborn, R., Hegseth, E., Falk-Petersen, S., Hoel, A. H., Eds.; Springer: Berlin, Germany, 2007; pp. 147–166.

Haq, S. M. Nutritional Physiology of *Metridia lucens* and *M. longa* from the Gulf of Maine. *Limnol. Oceanogr.* **1967**, *12*, 40–51.

Jaschnov, V. A. Order Copepoda. In *Guide to the Fauna and Flora of Northern Seas of the USSR*; Gaevskaya, N. S., Ed.; Soviet Nauka Press: Moscow, Russia, 1948; pp. 183–215.

Markhaseva, E. L. Calanoid Copepods of the Family Aetideidae of the World Ocean. *Proc. Zool. Inst. Russ. Acad. Sci.* **1996**, *268*, 1–331.

Mauchline, J. The Biology of Calanoid Copepods. In *Advances in Marine Biology*; Blaxter, J. H. S., Southward, A. J., Tyler, P. A., Eds.; Academic Press: Oxford, UK, 1998; Vol. 33, pp. 1–710.

Mumm, N. On the Summer Distribution of Mesozooplankton in the Nansen Basin, Arctic Ocean. *Ber. Polarforsch.* **1991**, *92*, 1–146.

Pomerleau, C.; Sastri, A. R.; Beisner, B. E. Evaluation of Functional Trait Diversity for Marine Zooplankton Communities in the Northeast Subarctic Pacific Ocean. *J. Plankton Res.* **2015**, *37*, 712–726.

Razouls, C.; Desreumaux, N.; Kouwenberg, J.; de Bovée, F. Biodiversity of Marine Planktonic Copepods (Morphology, Geographical Distribution and Biological Data); Sorbonne University, CNRS: Paris, France, 2025. Available online: http://copepodes.obs-banyuls.fr/en (accessed on 23 July 2025).

Richter, C. Regional and Seasonal Variability in the Vertical Distribution of Mesozooplankton in the Greenland Sea. *Ber. Polarforsch.* **1994**, *154*, 1–90.
